# Effects of Estrogens and Estrogenic Disrupting Compounds on Fish Mineralized Tissues

**DOI:** 10.3390/md12084474

**Published:** 2014-08-15

**Authors:** Patricia I. S. Pinto, Maria D. Estêvão, Deborah M. Power

**Affiliations:** 1 CCMAR—Centre of Marine Sciences, University of Algarve, Campus de Gambelas, Faro 8005-139, Portugal; E-Mails: mestevao@ualg.pt (M.D.E.); dpower@ualg.pt (D.M.P.); 2 School of Health, University of Algarve, Av. Dr. Adelino da Palma Carlos, Faro 8000-510, Portugal

**Keywords:** endocrine disrupting compounds, endocrine disruption, estrogen receptor, fish, mineralized tissues

## Abstract

Estrogens play well-recognized roles in reproduction across vertebrates, but also intervene in a wide range of other physiological processes, including mineral homeostasis. Classical actions are triggered when estrogens bind and activate intracellular estrogen receptors (ERs), regulating the transcription of responsive genes, but rapid non-genomic actions initiated by binding to plasma membrane receptors were recently described. A wide range of structurally diverse compounds from natural and anthropogenic sources have been shown to interact with and disrupt the normal functions of the estrogen system, and fish are particularly vulnerable to endocrine disruption, as these compounds are frequently discharged or run-off into waterways. The effect of estrogen disruptors in fish has mainly been assessed in relation to reproductive endpoints, and relatively little attention has been given to other disruptive actions. This review will overview the actions of estrogens in fish, including ER isoforms, their expression, structure and mechanisms of action. The estrogen functions will be considered in relation to mineral homeostasis and actions on mineralized tissues. The impact of estrogenic endocrine disrupting compounds on fish mineralized tissues will be reviewed, and the potential adverse outcomes of exposure to such compounds will be discussed. Current lacunae in knowledge are highlighted along with future research priorities.

## 1. Introduction

**Figure 1:**
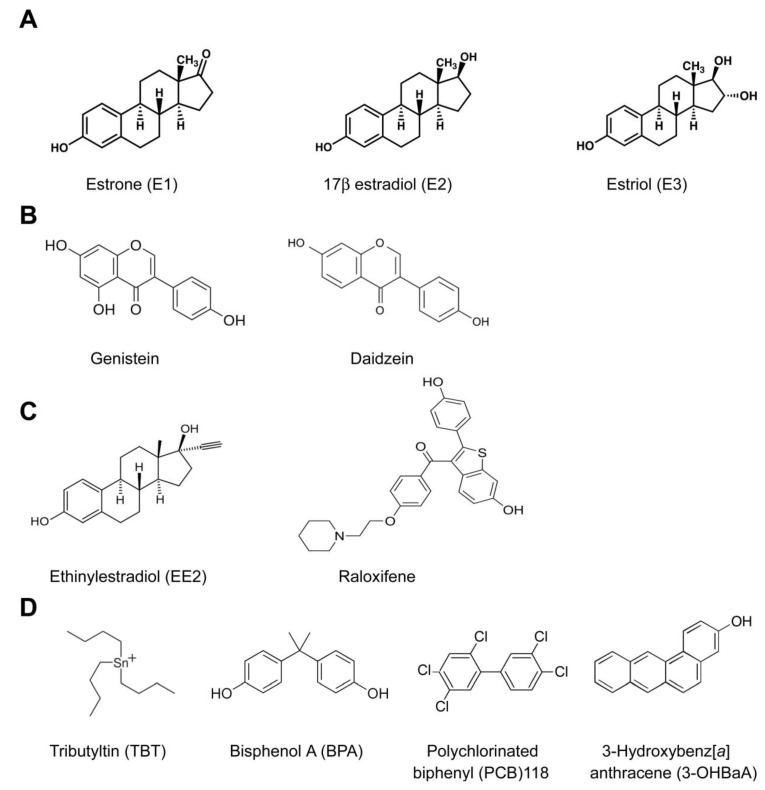
Examples of the chemical structure of typical estrogenic compounds, including: (**A**) natural animal estrogens; (**B**) natural plant estrogenic compounds (phytoestrogens); and synthetic estrogenic compounds with pharmaceutical (**C**) or other applications (**D**). The compounds were selected to show the diversity of chemical structures reported to possess estrogenic effects, and compounds with reported effects in fish mineralized tissues were preferentially selected (see Section 7).

Estrogens are ubiquitous, small steroid compounds that function as hormones mainly in vertebrates. The estrogen system in vertebrates is remarkably well conserved and includes steroidogenic enzymes involved in estrogen synthesis; the estrogens estrone, estriol and 17β-estradiol (E_2_, the most abundant and potent natural estrogen) (see Figure 1A), and estrogen receptors [1]. Estrogens are key regulators of physiological changes associated with reproduction in both sexes and also regulate many other important physiological processes, including immune function and mineral homeostasis [2,3,4].

A wide range of compounds have now been identified that affect the function of the estrogen system and are classified as endocrine disrupting compounds (EDCs), which are “exogenous substances or mixtures that alter the function(s) of the endocrine system and cause adverse health effects in an intact organism or its progeny” [5]. EDCs may act at several different levels, and the best studied actions are those in which compounds bind to estrogen receptors (ERs) and mimic or block normal estrogenic actions; however, other, less studied disruptive actions include alterations in receptor or hormone availability (affecting their synthesis, transport, metabolism and excretion), and other more recently identified mechanisms of action include disruption caused by binding to other receptors [6,7,8].

Estrogenic EDCs are structurally diverse compounds from multiple sources (see Figure 1 for examples) that have estrogenic and/or anti-estrogenic activities, although they may also affect other endocrine systems. Sources of estrogenic EDCs include natural estrogens produced by plants (phytoestrogens), fungi (mycoestrogens) and cyanobacteria, synthetic therapeutic drugs (e.g., raloxifene) and numerous synthetic compounds mainly used in industry and agriculture (e.g., polychlorinated biphenyls (PCBs), organochlorine pesticides, phthalate plasticizers or dioxins) [9,10].

Many EDCs are of anthropogenic origin and have been accumulating in the aquatic environment for decades, and their lipophilic and persistent nature means that they bioaccumulate and/or biomagnify in marine organisms [6,7]. Aquatic vertebrates, such as fish, are particularly affected by aquatic anthropogenic contaminants; exposure can be lifelong and through multiple routes, including the skin and gills or through feeding on contaminated sediments or organisms and bioaccumulation is frequent [11,12]. Aquatic contaminants can compromise reproduction, development, immune response and other physiological processes, which can ultimately affect the survival of fish [10,13,14]. In addition to the direct impact of aquatic contaminants on fish populations, the ecological importance of fish means that they also indirectly affect the environment and, when eaten by humans and wildlife, pose a health risk and negatively impact the economics of fisheries and aquaculture.

The estrogenicity of EDCs have mostly been evaluated in relation to their binding and/or activation of intracellular ERs, which regulate many of estrogens’ actions in target cells [7,15]. The adverse outcomes of exposure to estrogenic EDCs have mainly been evaluated in relation to reproductive functions or tissues (e.g., induction of hepatic vitellogenin production, reduced gonadal growth, male gonad feminization, altered sex ratios) [5,10,15]. In contrast, the impact of EDCs on non-reproductive tissues is largely unexplored [7,16]. From fish to mammals, mineral homeostasis is regulated by estrogens, and mineralized tissues are estrogen targets [1], which makes them also a target for endocrine disruption. 

The present review compares the effects and diverse mechanisms of action of estrogens on fish mineralized tissues and the better studied mammalian bone. Evidence for estrogen responsiveness and endocrine disruption will be reviewed for fish mineralized tissues. The possible mechanisms of action and the impact of exposure to estrogenic EDCs on fish health and survival is discussed, in relation to the physiological importance of the skeletal system.

## 2. Mineralized Tissues and Mineral Homeostasis

The skeleton has a well-recognized role in support, protection, locomotion and mineral homeostasis across vertebrates. Bone is a dynamic tissue maintained by continuous cycles of formation and resorption, mediated, respectively, by osteoblasts (OSB) and osteoclasts (OSC). Mammalian bone also contains cells embedded in the matrix, the osteocytes, that are crucial for the detection and response to mechanical loading, regulating bone remodeling and repair. Recently, mammalian bone has also been proposed to function as an endocrine organ that can influence reproduction and energy metabolism [17].

The fish skeleton consists of an articulated endoskeleton, like in mammals, but they also contain an exoskeleton formed by mineralized appendages, the fish scales. All fish mineralized tissues contain OSC and OSB that are thought to have the same role as in mammals. Fish bone is classified as acellular, because it lacks osteocytes [18], although salmonids and cyprinids are an exception, as they possess matrix dwelling osteocytes in some elements of the skeleton and, therefore, are considered to have cellular bone [19]. Due to the aquatic environment inhabited by fish, the skeleton is less exposed to mechanical loading; nonetheless, skeletal turnover and homeostasis are just as important as in mammals. Skeletal anomalies are a major concern in fish farms, as they can compromise fish survival and their economic value.

Mineral homeostasis requirements and regulation differ between mammals and fish and between marine and freshwater fish. In terrestrial vertebrates, the bone serves as a reservoir of minerals, as the only available source is the diet, and so, if there is an increased need for calcium (Ca), phosphorus (P) or other minerals, bone can be mobilized [20]. In marine species of fish, an abundant source of Ca is present in the environment, and food is only essential to meet the demand for P. In contrast, in freshwater fish species, Ca ions are in poor supply in the surrounding environment, and the diet contributes to meet Ca and P requirements, as occurs in mammals. If Ca becomes limiting, it can compromise reproduction, growth and development, leading to the mobilization of both Ca/P from mineralized tissues. In fish, the scales appear to play an important role in Ca/P homeostasis and also function as a physical barrier and improve hydrodynamics [21]. However, an important aspect to bear in mind when considering fish is the vast number of species that exist (over 30,000) and their diversity of habitats and adaptations. This means that caution is required when making generalizations between fish species and even more so in relation to the results obtained in other vertebrates, such as mammals. More studies of skeletal homeostasis and endocrine disruption are required in a greater diversity of fish, and it remains to be established if the recent novel role of bone in energy homeostasis in mammals also occurs in fish.

## 3. Estrogen Actions in Mineralized Tissues

Estrogens regulate mineralized tissues physiology and mineral homeostasis across vertebrates [1], together with other hormones. In mammals, E_2_ protects the skeleton by favoring differentiation and increasing the life span of OSB, by inducing bone mineralization and by decreasing the formation, activity and life span of OSC. Thus, in mammals, estrogens contribute to the development and maintenance of bone mass, in both males and females [22,23,24,25,26].

In fish, E_2_ induces an increase in plasma Ca levels and a decrease in scale Ca content in periods of increased demand, such as vitellogenesis in females [27,28,29,30]. This hypercalcemic effect appears to be mediated by several mechanisms, including an increase in Ca influx from the environment [2,31] and stimulation of Ca mobilization from mineralized tissues, especially the scales [32,33,34]. Indeed, in the scales of several marine and freshwater fish species, E_2_ increases the activity of tartrate-resistant acid phosphatase (TRAP), a marker of osteoclasts and scale resorption [33,34,35,36,37,38]. The results of such studies suggest that E_2_-induced Ca mobilization is especially increased in scales [39], which is in line with the protective role of E_2_ on mammalian bone. The effects of E_2_ on the fish endoskeleton remains to be fully characterized.

In goldfish (*Carassius auratus*, a freshwater teleost) and wrasse (*Pseudolabrus sieboldi*, a marine teleost), E_2_ also increased osteoblast activity (measured by the activity of the OSB marker, alkaline phosphatase, ALP) [37,40], suggesting that estrogens could affect both mineralization and mobilization in fish scales, two processes that are often coupled in mammalian bone [22]. Furthermore, exposure of early-life-stage mosquito fish to E_2_ causes a change in hemal spine morphology indicative of delayed development [41], while in E_2_ treated adult sea bream (*Sparus auratus*), the proteome of regenerating skin and scales indicates that the process is accelerated [42]. However, further studies are required to establish in more detail and in a greater number of species the effects of E_2_ on fish bone/scale Ca content and turnover and if this differs across the reproductive cycle or in relation to environmental or internal cues.

## 4. General Mechanisms of Estrogenic Action

The actions of estrogens are mostly mediated by specific intracellular receptors, the ERs, which may be located in the cytoplasm, the nucleus or in other organelles [7,43,44]. While most vertebrates have duplicate ER genes (α and β), the teleost-specific whole genome duplication means that most have at least three ER isoforms, one ERα and two ERβs (reviewed by [45]). The ERs in teleost fish have been detected in the nucleus and cytoplasm [46,47,48].

**Figure 2:**
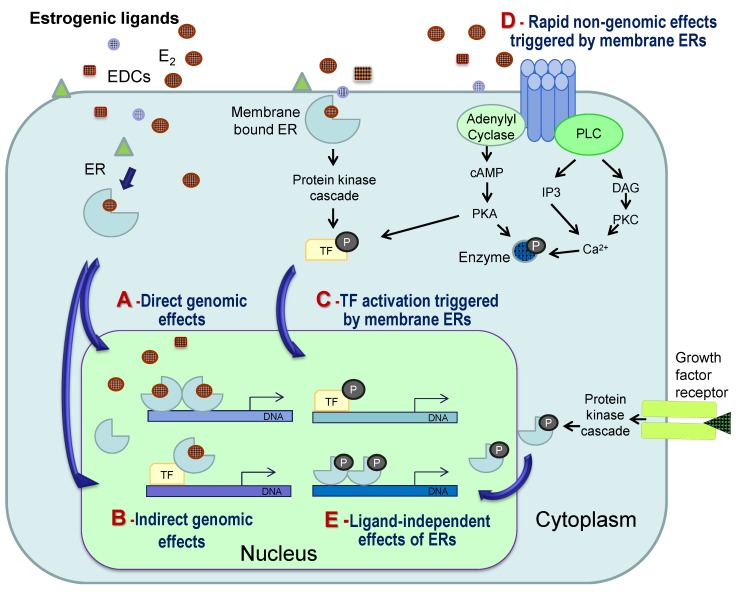
Simplified scheme presenting the possible mechanisms involved in the cellular actions of natural estrogens (e.g., 17β-estradiol, E_2_) and estrogenic endocrine disrupting compounds (EDCs). In the classical mode of action (**A**), an estrogenic ligand binds and activates intracellular estrogen receptors (in fish, ERα, ERβa or ERβb), which dimerize in the nucleus, bind to estrogen-response elements in the promoters of target genes and regulate their transcription, through the recruitment of a range of possible cell-specific co-regulators. Alternative mechanisms of action include: (**B**) indirect regulation of gene expression by interaction of ligand-bound ERs with other transcription factors (TF); (**C**,**D**) estrogen actions initiated by binding to membrane receptors (ERs or G-protein coupled receptors, such as the GPER) and activation of protein kinase cascades or alterations in the levels of secondary messengers, resulting in (**C**) the activation of transcription factors that regulate gene expression or (**D**) rapid non-genomic effects, such as the activation of specific enzymes. While genomic actions can take hours to days, non-genomic effects occur in seconds or minutes. In addition, ERs can be activated and regulate gene expression in a ligand-independent manner (**E**) through phosphorylation (P) in response to growth factor binding to their membrane receptors. Natural estrogens may compete with several EDCs (represented by different colors and shapes) for multiple receptors and pathways, resulting in a complex response that depends on the cellular context in terms of receptors and interacting proteins and, thus, may differ between tissues and circumstances. cAMP, cyclic AMP; PKA, protein kinase A; PLC, phospholipase C; IP3, inositol 1,4,5-triphosphate; DAG, diacylglycerol; PKC, protein kinase C. Adapted from [50].

In the classical model of estrogen actions (Figure 2A), ERs regulate gene expression in the nucleus of target cells by binding to estrogen-response elements (ERE) in the promoters of target genes [49,50]. Estrogens (as well as EDCs) can also act via alternative pathways (see Figure 2B–D for details), and studies in mammals reveal the indirect regulation of gene expression by interaction with other transcription factors (TFs) or by binding to membrane receptors, which may result in changes in gene expression or in rapid non-genomic effects, such as the activation of specific enzymes [7,50,51]. The nature of the receptors mediating membrane-initiated actions of estrogens remains controversial, but probably includes both classical ERs and their variants, as well as novel membrane receptors, such as the G protein-coupled estrogen receptor 1 (GPER, formerly known as GPR30), recently characterized in mammals and fish [52,53,54].

In fish, different mechanisms of E_2_ action have also been identified and include: (1) the direct genomic regulation of hepatic vitellogenin mRNA, a classical EDC biomarker, by intracellular ERs [55]; (2) disruption of ERα pathways by environmental contaminants through interaction with other transcription factors [56]; (3) indirect regulation of the luteinizing hormone β gene through ERα interaction with other TFs [57]; and (4) rapid GPER-mediated effects characterized in fish gonads [45,53].

## 5. Mechanisms of Estrogenic Action in Mineralized Tissues

The mechanism of action of E_2_ and other estrogenic compounds on the mammalian skeleton have been the focus of several recent reviews [22,23,58,59,60]. These appear to be complex and involve the interaction of E_2_ with other signaling pathways important for skeletal development and homeostasis that are activated by other hormones and factors, such as cytokines (e.g., interleukin-6, a major regulator of OSC formation and bone resorption), growth factors (e.g., insulin-like growth factor-I, IGF-I, regulator of bone linear growth) and parathyroid hormone (PTH) [23,58,61]. In addition, estrogenic effects on the skeleton include both direct actions on OSB, OSC, osteocytes and their precursor cells, as well as indirect effects, through regulating the crosstalk between cell types and released paracrine factors, which together orchestrate bone remodeling (for more detail, see [23,58,60]).

At the cellular level, direct actions via intracellular ERs that are localized in OSB, OSC and osteocytes [23,25,58,62,63] may include classical effects on gene expression [64] and also involve interaction with other TFs [23,58,65,66], and a number of bone estrogenic effects are mediated by non-nuclear actions of ERs or related receptors [58,67,68,69]. In addition, ligand-independent actions of ERs in mammalian bone were also recently identified and appear to differ between bone types [70]. There are thus multiple pathways and targets where EDCs can potentially interfere in bone, and although from fish to mammals there is evidence for the disruption of bone physiology by EDCs (see Section 7), the mechanisms involved are yet to be detailed [71].

**Table 1:** Detection of estrogen receptor isoforms in fish mineralized tissues.

Species	Tissue	Transcript/Protein and Detection Method	ERα	ERβa	ERβb	References
*Sparus auratus*	Endochondral bone (jaw)	Transcript (qRT-PCR)	√ (low)	√	√	[72]
*Sparus auratus*	Dermal bone (skull)	Transcript (RT-PCR)	-	√	√	[46]
*Sparus auratus*	Perichondral bone (vertebral centra)	Transcript (qRT-PCR)	√ (low)	√	√	[72,73]
*Sparus auratus*	Chondroid bone (gill arches)	Transcript (qRT-PCR)	√ (low)	√	√	[73]
*Sparus auratus*	Cartilage (Intervertebral disc)	Transcript (RT-PCR)	-	√	√	[46]
*S. auratus*; *Oreochromis mossambicus*; *Carassius auratus*; *Oncorhynchus mykiss*;	Scales	Transcript (RT-PCR); Protein (IHC)	√ (low)	√	√	[32,40,46]
*Sparus auratus*	Skin with scales	Transcript (qRT-PCR)	√ (low)	√	√	[42]

RT-PCR, reverse transcription polymerase chain reaction; qRT-PCR, quantitative RT-PCR; IHC, immunohistochemistry; low, low level of expression.

In fish, the mechanisms responsible for the effects of estrogens in mineralized tissues are mostly unexplored. Transcripts for the three fish ER isoforms were detected in different fish mineralized tissues (Table 1), with a higher abundance of the ERβa and βb isoforms. In the scales of sea bream and Mozambique tilapia (*Oreochromis mossambicus*), the three ER protein isoforms were detected in putative OSC [46], suggesting that the E_2_-induced Ca mobilization and increase in scale OSC activity [34,37] may result from direct actions in these cells. In contrast, fish ERs were not detected in scale OSB, despite the fact that E_2_ is able to alter fish scale OSB activity [37,40], raising questions about its mechanism of action.

In sea bream, transcript levels for ERβa were upregulated by E_2_ in both intact skin-scale and in regenerating skin after scale removal, while ERβb was downregulated by E_2_ in regenerating skin [42]. In addition, the selective ER modulator (SERM), raloxifene (an ER agonist in mammalian bone), upregulated both ERβa and βb in dentary bone and downregulated ERβa in vertebra [72]. Taken together, these studies suggest that estrogenic compounds most probably exert at least part of their actions on the fish skeleton or skin-scale through the duplicate ERβs (especially the βa isoform), rather than ERα.

In common with mammals, it appears that the expression, localization and regulation of different ER isoform in fish differ between different types of mineralized tissues [42,46,72] (Table 1), probably giving rise to different responsiveness to estrogens and to the potential for interaction or different actions between ER isoforms. The relative and tissue-specific role of ER isoforms in mediating estrogenic actions in fish mineralized tissues, as well as the expression and involvement of other possible players (e.g., ER variants, membrane ERs, other hormones or their receptors) will need further investigation. Recently, GPER transcripts were detected in sea bass (*Dicentrarchus labrax*) scales, indicating another possible mechanism of action for E_2_ and EDCs (author’s unpublished results, [74]).

Finally, evidence exists that indicates that the E_2_ effects on fish scales may also be in part mediated by other hypercalcemic factors, such as members of the parathyroid hormone (PTH)/PTH-related protein (PTHrP) family [75], shown to regulate scale OSC/OSB activities *in vitro* and reduce scale Ca content *in vivo* [35,76]. In fact, cross-talk occurs between E_2_ and PTH/PTHrP, and *in vivo* injections of a PTH/PTHrP receptor antagonist also partly inhibit the E_2_ stimulated hypercalcemic effect [77]. *In vitro* treatment of goldfish scales with E_2_ stimulates both OSB and OSC activities and upregulates mRNA expressions of IGF-I and cathepsin K, which are respectively OSB/OSC markers, and it will be interesting in the future to analyze the change in expression or activity of other paracrine factors in scales or different types of fish bone in response to E_2_ and other estrogenic compounds.

## 6. Estrogenic Endocrine Disruption

In the context of the diversity of mechanisms and receptors for estrogens, it is now clear that in a given cell context, the mode of action of a certain compound as an agonist, antagonist or a SERM (compounds with agonist or antagonist estrogenic actions depending on the tissue) depends not only on its structure, but also on tissue- and cell-specific factors (summarized in Table 2) [7,51]. In fish, the existence of an additional ER isoform relative to other vertebrates [45] adds another layer of complexity to the diversity of possible estrogenic ER-mediated mechanisms of action.

The most studied EDCs are estrogenic [7], and as previously indicated, there is a wide range of structurally diverse compounds from both natural and anthropogenic sources that have estrogenic effects and to which humans and wildlife may be exposed, via the diet or from the surrounding environment (examples are given in Section 1 and Figure 1) [6,78]. The four main factors may explain the high susceptibility of the estrogen system to disruption by diverse EDCs, namely:

(1) The high number of natural and anthropogenic chemical compounds with structural similarity to natural estrogens [78];
(2) The high promiscuity of ERs and the ability of their binding pocket to accommodate and be activated or repressed by a wide range of (structurally diverse) lipophilic compounds [51,79];
(3) The different levels of the estrogen system components that can be disrupted, including the enzymatic pathways involved in steroid biosynthesis and/or metabolism (still poorly explored), as well as the target cell final actions [13,80];
(4) The diversity of receptors, mechanisms and signaling pathways involved in the cellular action of estrogens, which are also targets for EDCs (see Section 4 and Figure 2) [7,51].

**Table 2:** Factors that influence the cellular response to an estrogenic EDC.

Factor		Influence
Structure of the chemical:		Determines binding to a given receptor and the resulting receptor conformation (agonist or antagonist-type)
Cellular context:	Diversity and functional characteristics of receptors	The expression, sub-cellular localization and functional characteristics of intracellular ERs, their variants or membrane ERs/GPERs determine the signaling pathways that are activated or repressed
	Diversity of coregulators	The cellular context in terms of the presence and levels of co-repressors and/or co-activators greatly influences cell-specific effects on an estrogenic ligand
	Diversity of other transcription factors	The diversity of other transcription factors influences the possibility of indirect actions on alternative genes

The importance of mineralized tissues for animal health and survival across vertebrates (Section 2), makes understanding the estrogenic and/or antiestrogenic effects of EDCs on this tissue a priority (Section 3), particularly taking into consideration the vast range of putative estrogenic EDCs and the fact that estrogen responsiveness is tissue-specific. However, until now, the vast majority of studies of estrogenic EDCs in fish have screened exclusively for disruptive effects on reproduction.

## 7. Estrogenic Endocrine Disruption in Mineralized Tissues

In tetrapods, exposure to environmental contaminants, particularly EDCs, is known to affect both OSB and OSC functions and bone characteristics. Moreover, in mammals, exposure to EDCs during the perinatal period may have an impact during adult life, while other effects may be a consequence of exposure at any time during life, and different effects have been reported for different bone types (reviewed by [71]). Several *in vivo* and *in vitro* EDC exposure studies carried out in mammals, amphibians and reptiles support the hypothesis that the disruption of cellular actions involved in bone formation and remodeling may have consequences for bone architecture, strength, density (mineral content) and characteristics (hardness and elasticity) [71,81,82].

However, relatively few studies (summarized in Table 3) have evaluated the impact of estrogenic EDC exposure on mineralized tissue in fish. The impact of these compounds was analyzed using *in vivo* and *in vitro* approaches and mostly using as endpoints OSB and OSC activity, plasmatic Ca levels, the incidence of bone anomalies and the expression of molecular markers associated with bone formation (receptor activator of the NF-κB ligand (RANKL) and IGF-I) and resorption (TRAP and cathepsin K).

Results suggest that some EDCs can have an impact on skeletal development, morphology and anomalies in fish, including the environmental contaminants, 17 alpha-ethynylestradiol (EE_2_), bisphenol A (BPA), 2,3,7,8-tetrachlorodibenzo-p-dioxin (TCDD), PCB 77 and the estrogen antagonist, ZM189,154 (ZM) [83,84,85,86]. However, morphological abnormalities may be insensitive indicators, since long exposure and high concentrations are required to have an overt effect [83], and they may also not be related to endocrine disruption [87]. Nonetheless, the exposure of fish to polluted water from sewage treatment plants with putative estrogenic effects caused detectable changes in hemal spine morphology [88].

The effects of several EDCs were observed on scale TRAP and ALP enzymatic activities, used respectively as markers for OSC and OSB activities [34,40,89]. Bisphenol A and two monohydroxylated polycyclic aromatic hydrocarbons (OHPAHs) suppressed both osteoclast and osteoblast activity [37,90], while tributyltin acetate (TBTA) did not affect OSC activity [91]; and PCB 118 increased it [92] (Table 3).

The mechanisms mediating these effects were not established, although the transcript abundance of OSB markers (IGF-I and RANKL) and OSC markers (TRAP and cathepsin K) was analyzed [37,90,92]. IGF-I transcript abundance was unaltered by exposure of goldfish scales to BPA, but was downregulated, together with cathepsin K, by 4-OHBaA, an OHPAH. In contrast, the exposure of goldfish scales to E_2_ upregulated both transcripts [37], and PCB 118 upregulated cathepsin K, TRAP and RANKL [92]. Several of these contaminants have previously been shown or predicted to bind and/or activate fish ERs (e.g., [87,93,94,95]), suggesting that part of the effect of these EDCs is via a direct action on intracellular ER expression in fish scales.

The diverse methodology and limited endpoints measured, such as changes in enzyme activity, morphology or targeted gene transcripts, together with the diversity of EDC doses tested in different freshwater and marine fish species, makes it difficult to compare the outcomes of studies. A comparison of the impact of putative estrogenic EDCs contaminating aquatic environments, using a common methodology and a range of endpoints, would facilitate the understanding of their mechanisms of action on OSB and OSC and their precursor cells. In fact, recent recommendations from the scientific community and public organizations have highlighted the need to identify molecular markers for xenoestrogen actions on target tissues, so that they can be included in risk assessment, particularly of wildlife [13,16,78,96,97,98].

It is clear that, even if only the marine environment is considered, when screening EDCs for their impact on fish mineralized tissue and on E_2_-regulated processes, several factors need to be taken into consideration:

(1) The diverse fish species of ecological and commercial interest, in the wild or reared in aquaculture units, which may have different responses;
(2) The endpoints that should be evaluated to assess EDC effects and elucidate the mode of action;
(3) The tissue-specific responses to EDCs. For example, scales are proposed to be a preferential site for E_2_-induced Ca mobilization compared to bone [33,39], and they are directly exposed to the aquatic environment and for these reasons have been preferentially studied as an EDC target tissue (Table 1). However, the estrogenic EDCs impacts on the fish endoskeleton and different bone types (endochondral, dermal, chondroid) need to be studied, as they respond differently to estrogenic compounds [46,72];
(4) The number of contaminants present in the aquatic environment both alone (in different concentrations) or in complex mixtures that may have an estrogenic disrupting action.

In addition, it is likely that the compounds that disrupt bone metabolism in mammals [71] may also have disruptive estrogenic actions in fish bone and/or scales, when the conservation of the estrogenic axis in mineralized tissue between fish and mammals is taken into consideration. Moreover, many EDCs already have identified disruptive actions on fish reproductive tissues and bind and/or transactivate fish ERs (e.g., [16,78,87,94,99,100,101,102,103]), and together, these may be good starting candidates to investigate the impact of EDCs in fish mineralized tissues.

The study of EDC effects represents an endless, but necessary task to prevent future damage to fish species, wildlife and human welfare in general. More specifically, estrogenic disruption of mineralized tissues may have a wide range of consequences, since an increase in skeleton anomalies, modified bone density and mineral homeostasis may impact on swimming and capacity to capture prey and escape from predators. In addition, the protective role of scales and their contribution to mineral (mainly Ca/P) homeostasis may be compromised and affect wild and cultured fish fitness and productivity in aquaculture units. An indirect impact of EDC-induced modifications in mineralized tissue that has so far not been considered is the effect of modified Ca availability on reproductive success, and this may reinforce the impact of EDCs on the reproductive system.

There are several EDC testing programs being implemented by non-governmental advisory bodies throughout the world in order to identify or predict the endocrine disruptive effects of compounds and to prioritize the screening of environmental EDCs [87,97,98,104]. Most of these tests include *in vivo* assays with fish, since this is a group with clearly documented adverse impacts of EDCs. Testing tends to use small fish species, easily reared and with a short life cycle and is mainly focused on reproductive endpoints, e.g., the fish short-term reproduction assay (which evaluates the effects of exposure on secondary sexual characteristics, vitellogenin levels, fecundity and gonad histopathology in adult fish) or the fish sexual development test (which evaluates the effects of exposure during development on vitellogenin levels and sex ratio) [11,16,97,105,106].

The acknowledged limitations of current tests are their failure to cover mechanisms of action, the need to sacrifice fish and their time-consuming and/or expensive character [16,87,96].

Recent trends are to implement alternative *in vitro*, short-term assays to detect estrogenic endocrine disruption. For the risk assessment of putative estrogenic EDCs, recommended tests include ER ligand binding, cell-free assays, yeast reporter gene assays, cell line reporter assays, primary cell culture, as well as embryo and organ culture tests for many fish species [87] However, the existing assays are uninformative about EDC effects on mineralized tissues, and data about the adverse effects on these tissues are still limited. Future studies to cover a wider range of estrogenic EDCs, doses and fish species are required and could benefit from the inclusion of rapid test methods for mineralized tissues.

**Table 3:** Selected examples of the reported effects of estrogenic disrupting compounds on fish mineralized tissues.

Species	Compound	Effective Dose	Exposure Type and Period	Endpoint	Effect	Reference
*Pimephales promelas*	17α-ethynylestradiol (EE2)	0.1 to 100 μg/L	*In vivo*, from 24 hpf to 25–26 dph	Degree of skeletal development; spinal abnormalities	Modified skeletal developmental; vertebral malformations in up to 62% of fish	[86]
	Bisphenol A (BPA)	1000 μg/L			No impairment of skeletal development or vertebral malformations; decreased developmental score	
*Fundulus heteroclitus*	17α-ethynylestradiol (EE2)	1000 to 10,000 ng/L 10 and 10,000 ng/L	*In vivo*, first 25 or 60 day of life	Skeletal and soft tissue abnormalities	Increased % of abnormal fish; increased number of abnormalities per fish	[83]
	ZM189,154(ZM)				Increased incidence of scoliosis; decreased overall incidences of abnormal fish and lordosis	
*Gambusia holbrooki*	Sewage (two sewage treatment plants)	n.a.	n.a.	Hemal spines morphology	Modified hemal spines with one sewage source	[88]
*Carassius auratus*	Bisphenol A (BPA)	10^−6^ to 10^−5^ M	*In vitro*, scale assay (6 h)	TRAP and ALP activity; transcript expression	Suppressed OSB and OSC activity; no changes in IGF-I expression	[90]
*Carassius auratus* (freshwater); *Girella punctata* and *Pseudolabrus sieboldi* (marine)	Tributyltin acetate (TBTA)	10^−9^ to 10^−5^ M	*In vitro*, scale assay (6 h)	TRAP and ALP activity	Inhibits OSB activity; no effect on OSC activity	[91]
*Carassius auratus* (freshwater); *Pseudolabrus sieboldi* (marine)	3- and 4-OHBaA	10^−7^ to 10^−5^ M	*In vitro*, scale assay (6 and 18 h)	TRAP and ALP activity; transcript expression	Inhibited OSB and OSC activities 4-OHBaA down-regulated cathepsin K and IGF-I expression	[37]
*Carassius auratus*	Polychlorinated biphenyl (PCB 118)	100 ng/g BW 0.0025–2.5 ppm	*In vivo*, intraperitoneal injection (2 days) *In vitro*, scale assay (6 and 18 h)	TRAP and ALP activity in scales Ca level in plasma transcript expression	Increased OSC activity; hypercalcemia; increased OSC and OBS activity; upregulated cathepsin K, TRAP and RANKL expression	[92]
*Sparus auratus*	Raloxifene	3.33 mg/kg BW	*In vivo*, intraperitoneal injection (6 days)	Ca level in plasma balance; transcript expression in dermal and perichondral bone	No change in Ca levels; downregulation of genes related to bone formation and resorption in vertebra (perichondral bone)	[72]

ALP, alkaline phosphatase; BW, body weight; dph, days post-hatch; hpf, hours post-fertilization; IGF-I, insulin-like growth factor I; OSB, osteoblasts; OSC, osteoclasts; RANKL, receptor activator of the NF-κB ligand; TRAP, tartrate-resistant acid phosphatase; 3-OHBaA, 3-hydroxybenz[a]anthracene; 4-OHBaA, 4-hydroxybenz[a]anthracene.

Non-invasive, less expensive and more rapid tests are essential, and the fish scale assay may represent an interesting option that can be used simultaneously for many different fish species, but it needs to be optimized, validated and established as a high throughput test. The use of recent technologies based on genomics, transcriptomics or proteomics will provide insight into the mechanisms of estrogens’ actions [14] and, if applied to fish mineralized tissues, could be used to identify possible biomarkers representing different endpoints of EDC exposure.

## 8. Conclusions

Estrogens regulate calcified tissue physiology and mineral homeostasis across vertebrate, and despite the general conservation of OSB and OSC functions, their regulation by estrogens appears to differ between mammals and fish, presumably as a consequence of specific adaptations of their bone structure and metabolism.

Fish mineralized tissues express intracellular ERs, although their expression and regulation appears to depend on the tissue, and preliminary results suggest that fish scales also express plasma membrane estrogen receptors. Further studies are required to localize receptors in different fish bone tissues and cell types and to detail their regulation by estrogens throughout the reproductive season and in response to environmental factors or other hormones. The expression of ERs in fish mineralized tissues indicates that they may be targets for the direct effects of E_2_ and the potential disruption by EDCs present in the environment or in the diet, and candidate compounds for testing this include those with an effect on mammalian bone or on fish reproductive tissues. Until now, relatively few studies have demonstrated the effects of EDCs on fish mineralized tissues, and studies of endocrine disruption on the teleost scale (which mainly used as endpoint OSC/OSB activities, Ca levels or morphology) are limited in the number of endpoints, species, compounds, doses and possible mechanisms investigated. In addition, the diversity of fish species (over 30,000) and the species-specific response of fish bone to E_2_ mean generalizations about the impact of EDCs are of limited usefulness.

Further investigation of the action of EDCs on fish mineralized tissue is required and should include, amongst other things:

(1) Screening of a far greater number of EDCs, including endpoints, such as the assessment of enzyme activities, gene expression, proteome changes and gene networks and cellular pathways;
(2) Characterization of tissue-specific responses (e.g., bone type and bone *versus* scale);
(3) Establishment of species-specific, season-specific and age-specific responses;
(4) Determination of the impact of estrogenic disruption on fish health and survival.
